# Effects of Different Treatment Methods of Dried Citrus Peel (*Chenpi*) on Intestinal Microflora and Short-Chain Fatty Acids in Healthy Mice

**DOI:** 10.3389/fnut.2021.702559

**Published:** 2021-07-26

**Authors:** Yujiao Qian, Zhipeng Gao, Chen Wang, Jie Ma, Gaoyang Li, Fuhua Fu, Jiajing Guo, Yang Shan

**Affiliations:** ^1^Longping Branch, Graduate School of Hunan University, Changsha, China; ^2^International Joint Lab on Fruits & Vegetables Processing, Quality and Safety, Hunan Key Lab of Fruits & Vegetables Storage, Processing, Quality and Safety, Hunan Agriculture Product Processing Institute, Hunan Academy of Agricultural Sciences, Changsha, China; ^3^College of Animal Science and Technology, Hunan Agricultural University, Changsha, China

**Keywords:** *chenpi* powder, *chenpi* decoction, intestinal microbiota, short chain fatty acids, different treatment methods

## Abstract

*Chenpi* is a kind of dried citrus peel from *Citrus reticulata*, and it is often used as traditional Chinese medicine to treat dyspepsia and respiratory tract inflammation. In this study, to determine which way of *chenpi* treatment plays a better effect on the prevention of obesity in healthy mice, we conducted 16S ribosomal RNA (rRNA) gene sequencing for intestinal microbiota and gas chromatography-mass spectrometry detector (GC/MSD) analysis for short-chain fatty acids (SCFAs) of female rats fed with either *chenpi* decoction or *chenpi* powder-based diet (*n* = 10 per group) for 3 weeks. C*henpi* powder (CP) group significantly reduced abdominal adipose tissues, subcutaneous adipose tissue, and the serum level of total triacylglycerol (TG). At a deeper level, *chenpi* powder has a better tendency to increase the ratio of *Bacteroidetes* to *Firmicutes*. It alters the *Muribaculaceae* and *Muribaculum* in intestinal microbiota, though it is not significant. The concentrations of acetic acid, valeric acid, and butyric acid increased slightly but not significantly in the *CP* group. *Chenpi* decoction just reduced perirenal adipose tissues, but it shows better antioxidant activity. It has little effect on intestinal microbiota. No differences were found for SCFAs in the *chenpi* decoction (CD) group. The results indicated that *chenpi* powder has a better effect in preventing obesity in mice. It can provide a basis for the development of functional products related to *chenpi* powder.

## Introduction

Dried citrus peel (*chenpi*) is the mature dry pericarp of *Citrus reticulata*. As a traditional Chinese medicine, it has a good effect on treating dyspepsia and improving respiratory tract inflammation. *Chenpi* contains many active components, such as essential oil (1), flavonoid (2), pectin (3), insoluble fiber (4), and so on. Citrus peel essential oils may ameliorate hypercholesterolemia and hepatic steatosis by modulating lipid and cholesterol homeostasis, and most of them have good antimicrobial and antioxidant activities (5, 6). Polymethoxyflavones, a kind of flavonoid from citrus peel, have anti-obesity, anti-hyperglycemic, and antiviral activities; meanwhile, it may effectively prevent the progression of metabolic syndrome (7–10). Pectin polysaccharide has *in vitro* intestinal immunomodulatory activity (11). In addition to the abovementioned active substances, pure *chenpi* powder also contains a large amount of dietary fiber. The composition and activity of intestinal microbiota and the production of short-chain fatty acids (SCFAs) were affected by dietary fiber (12). Meanwhile, the production of SCFAs (in particular, acetate, propionate, and butyrate) is closely related to intestinal health and function (13).

Intestinal microbiota are microorganisms colonized in the human digestive tract, which is closely related to age, obesity, and inflammation (14–16). In recent years, the study on intestinal microbiota is a hot spot. Diet has different effects on intestinal microflora. More and more evidence shows that intestinal microflora is closely related to metabolism, host gene expression, and other factors (17–19). *Chenpi* has been proven to have a modulation effect on the composition of intestinal microbiota species, the abundance of microbiota, fecal SCFAs, intestinal barrier function, and gastrointestinal inflammation (20–22).

Obesity as a thorny issue worldwide is caused by many factors. Obesity can cause a series of complications, such as hypertension, hyperlipidemia, metabolic diseases, and increasing organ burden (23, 24). Several studies have observed the effects of extracts or natural products on intestinal microorganisms, SCFAs, glucose metabolism, and body weight of healthy mice model (25, 26). Looking for natural products that can alleviate and treat obesity is a healthy and safe method. Although there are some studies on the effect of reducing weight and lipid of *chenpi*, there is no study on which way of *chenpi* treatment can play a better effect. In this experiment, we observed the effect of the *chenpi* on healthy mice. Traditionally, *chenpi* was infused with boiling water to extract their effective components such as “decoction.” In this study, we added *chenpi* to the normal diet of mice in two forms, both *chenpi* decoction and *chenpi* powder. This study aimed to investigate the modulation effect of two different types of *chenpi* on the accumulation of adipose, intestinal microbiota, antioxidant capacity, and SCFAs to unvail their potential application for obesity prevention, which may also provide a basis for the use of *chenpi* as a kind of anti-obesity food in the food industry.

## Materials and Methods

### Mice and Housing

Forty four-week-old C57BL/six female mice (Tianqin Biotechnology Company, Changsha, China) were housed in a controlled room with a 12 h/day lighting cycle during the experimentation. Food and drinking water were freely available to mice. Following 1 week of acclimation, mice (*n* = 10) were randomly grouped to control (C), *chenpi* decoction (CD), control powder (P), *chenpi* powder (CP). They were all provided with a normal diet. The normal diet contained 54.9% corn, 5.6% casein, 18% soybean meal, 6.5% beer yeast, 0.7% lard, 0.8% bean oil, 0.5% salt, 1.4% fishmeal, and 1% premixture. The difference between granulated (C) and powder (P) groups is whether granulation is carried out. In the CD group and CP group, *chenpi* decoction and *chenpi* powder, respectively, were added to the normal diet. The body weight, food intake, and water intake were recorded once a week. After 3 weeks of administration, blood samples were collected by orbital bleeding. Liver, abdominal adipose tissues, subcutaneous adipose tissues, and perirenal adipose tissues were weighed and collected. Fecal samples were collected by 16S ribosomal RNA (rRNA) sequencing and analysis of SCFAs. The experimental protocol was approved by the Animal Care and Use Committee of Hunan Agricultural University.

### Preparation of *Chenpi* Decoction and *Chenpi* Powder

*Chenpi* was purchased from Jiangmen Xinhui tangerine peel village market limited company, Guangdong Province. The variety of *chenpi* is red *Pericarpium Citri Reticulatae*, which is made by traditional sunlight drying. According to the traditional decocting method, 10 g *chenpi* was crushed into a coarse powder and 200 ml of water was added and boiled over 95°C for 30 min. The filtrate was filtered out and then added 20 times of water to decoct again in the same way. The filtrate was combined, evaporated, and concentrated to 10 ml and stored at 4°C. The concentration of *chenpi* decoction was 1 g/ml. CD group were administered 0.2 ml/day *chenpi* decoction by gavage. The mice in the C group were given distilled water at the same time. After grinding and sieving, the *chenpi* powder was sealed in vacuum and stored at 4°C. The CP group were given 0.2 g/day *chenpi* powder in the diet.

### Histopathological Observation

Paraformaldehyde solution in 4% was used to fix adipose tissues. Then, they were dehydrated by ethanol solution, embedded, and prepared. The subcutaneous adipose tissue was stained with H&E. Images were obtained using a Nikon Eclipse E100 Upright optical microscope from Nikon Corporation, Japan (27).

### Biochemical Analysis

The serum concentration of total cholesterol (TC), total triacylglycerol (TG), high-density lipoprotein cholesterol (HDL-C), and low-density lipoprotein cholesterol (LDL-C) were determined by using Kehua biological automatic biochemical analyzer. Biochemical kits were purchased from Shanghai Kehua Bio-Engineering Co., Ltd (Shanghai, China) (28).

### Measurement of Hepatic Malondialdehyde (MDA) and Superoxide Dismutase (SOD) Levels

About 0.5 g of each liver tissue was homogenized in 4.5 ml frozen normal saline and then centrifuged and collected supernatant at 2,000 rpm for 10 min at 4°C for measurements. All these biochemical markers were measured using kits purchased from the Nanjing Jiancheng Bioengineering Institute (Nanjing, China). Coomassie Brilliant Blue was used to determine the concentration of protein (27). Each sample has a parallel sample.

### 16S Ribosomal RNA (rRNA) Gene Sequencing for Microbiota Profiling

Total genomic DNA was extracted from fecal samples and stored at −20°C using the DNA kit according to the instructions for 16S rRNA gene pyrosequencing. Paired-end sequencing was performed on the Illumina MiSeq platform (29). The V3-V4 regions were amplified using a specific primer with the barcode by thermocycler PCR system. In summary, α-diversity, β-diversity, and principal coordinate analysis (PCoA) were calculated and generated by Quantitative Insights Into Microbial Ecology (QIIME). The measurement was repeated three times for each sample. The online platform of Majorbio Cloud (http://www.majorbio.com/) was used to analyze data (30, 31).

### Detection of SCFAs

A total of 100 mg feces were dissolved in 0.9 ml water, then mixed, and then centrifuged at 13,200 g force for 10 min at 4°C. A 1 μl supernatant of each sample was injected into the inlet for gas chromatography-mass spectrometry detector (GC/MSD) analysis. The levels of acetic, propionic, butyric, valeric, isobutyric, and isovaleric acids in SCFAs were measured using 8890B-5977B GC/MSD (Agilent Technologies Inc. CA, USA) (32, 33). The measurement was repeated three times for each sample.

### Statistical Analysis

The statistical analyses were completed using IBM SPSS Statistics 26.0. The *t*-test was performed to determine the difference between groups. Values of *P* < 0.05 mean statistically significant.

## Results

### *Chenpi* Alleviates Accumulation of Adipose in Mice

**Figure 1:**
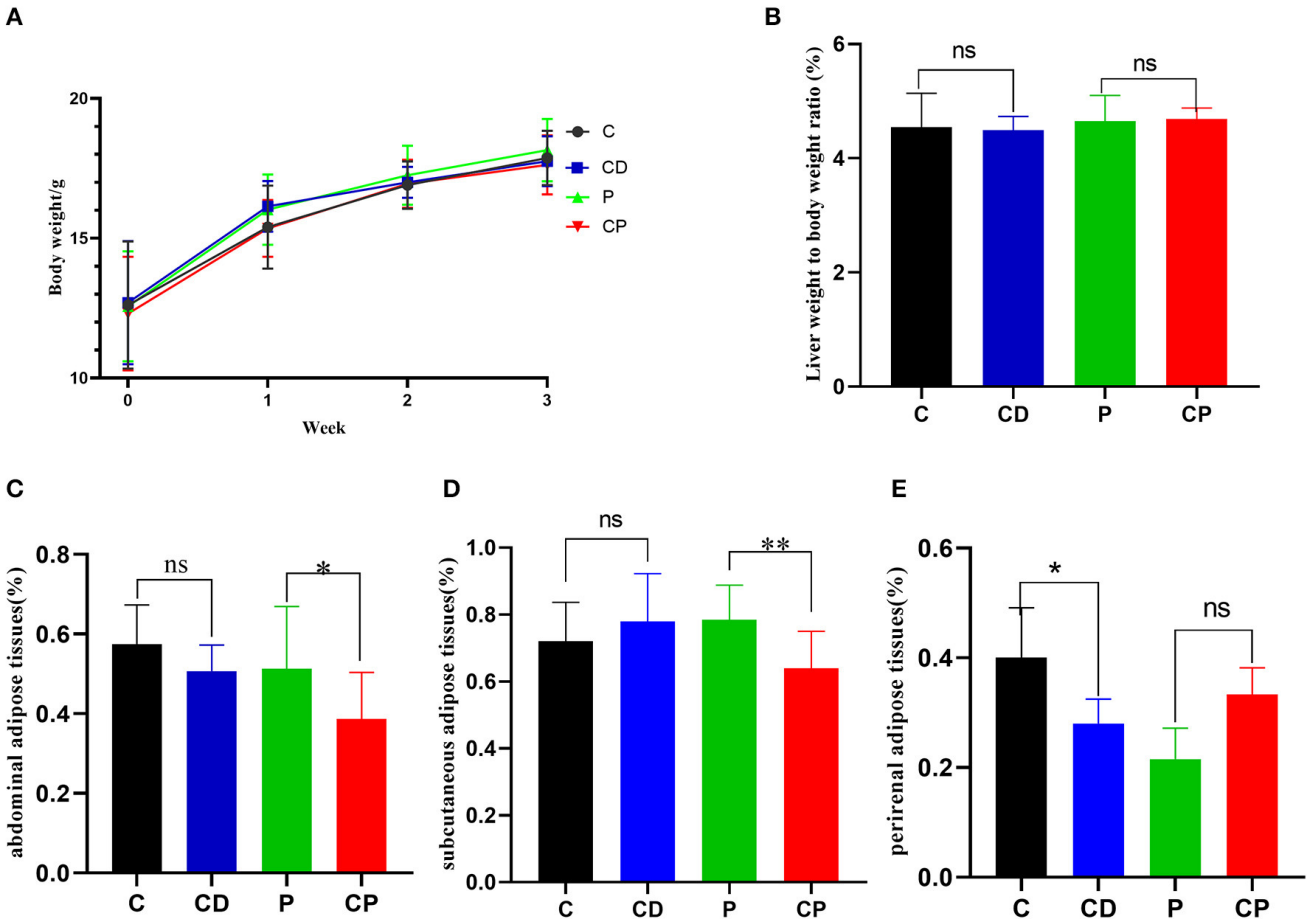
Chenpi alleviated the accumulation of adipose in mice (*n* = 9–10). **(A)** The body weight in 3 weeks (g); **(B)** the relative weight of liver to body weight; **(C)** abdominal adipose tissues to body weight ratio (%); **(D)** subcutaneous adipose tissues to body weight ratio (%); and **(E)** perirenal adipose tissues to body weight ratio (%). * *P* < 0.05; ** *P* < 0.01; and ns *P* > 0.05.

**Figure 2:**
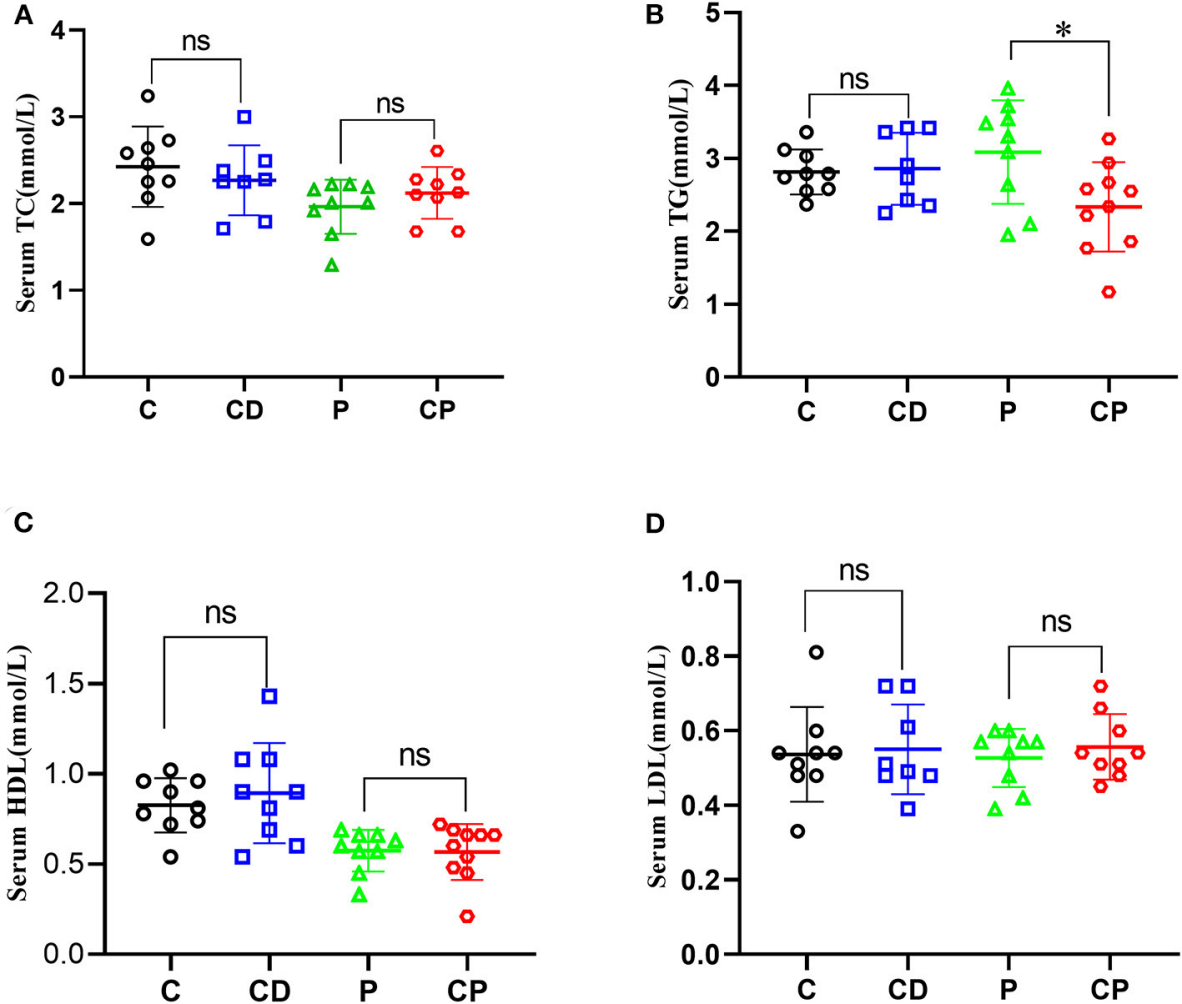
Chenpi adjusted serum concentrations of lipids. Concentrations of total cholesterol (TC) **(A)**, total triacylglycerol (TG) **(B)**, high-density lipoprotein cholesterol (HDL-C) **(C)**, and low-density lipoprotein cholesterol (LDL-C) **(D)** in serum (*n* = 8–10). * *P* < 0.05; ns *P* > 0.05.

**Figure 3:**
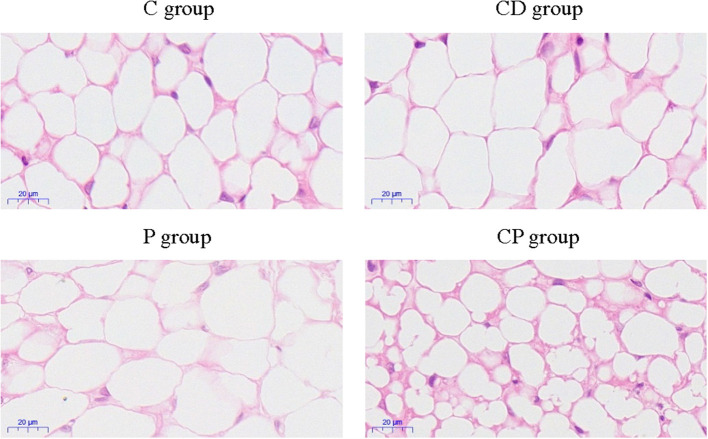
The observation of subcutaneous adipose tissues by H&E staining of four treatment groups (×400).

To determine the anti-obesity effect of *chenpi* on mice, body weight, liver, abdominal adipose tissues, subcutaneous adipose tissues, and perirenal adipose tissues were weighed. *Chenpi* treatment has a tendency to regulate body weight, but the difference was not significant (*P* > 0.05) (Figure 1A). Liver weight has basically no change in every group (Figure 1B). Weight of abdominal adipose tissues and subcutaneous adipose tissue was significantly reduced at 0.13 and 0.15% in the CP group compared with the P group (*P* < 0.05) (Figures 1C,D). Perirenal adipose tissues were significantly reduced in CD (*P* < 0.05) compared with the C group (Figure 1E). Serum concentrations of lipids were analyzed (Figure 2) to find out that *chenpi* powder can significantly reduce the serum level of TG by 24% compared to the P group (*P* < 0.05) (Figure 2B) but had no remarkable effect on the serum levels of TC, LDL-C, and HDL-C. These items showed no significant changes in the CD group compared to the C group. The histopathological observation of adipose tissues showed that the CP group exhibited a strong inhibitory effect on the enlargement of adipocytes compared with the P group, while the difference was not significant in other groups (Figure 3). To sum up, compared with *chenpi* decoction, supplementation of *chenpi* powder in the diet significantly alleviated accumulation of lipid and serum TG metabolism, and it reduced the relative weight of abdominal adipose tissue and subcutaneous adipose tissue.

### *Chenpi* Enhanced Antioxidant Capacity in the Liver

**Figure 4:**
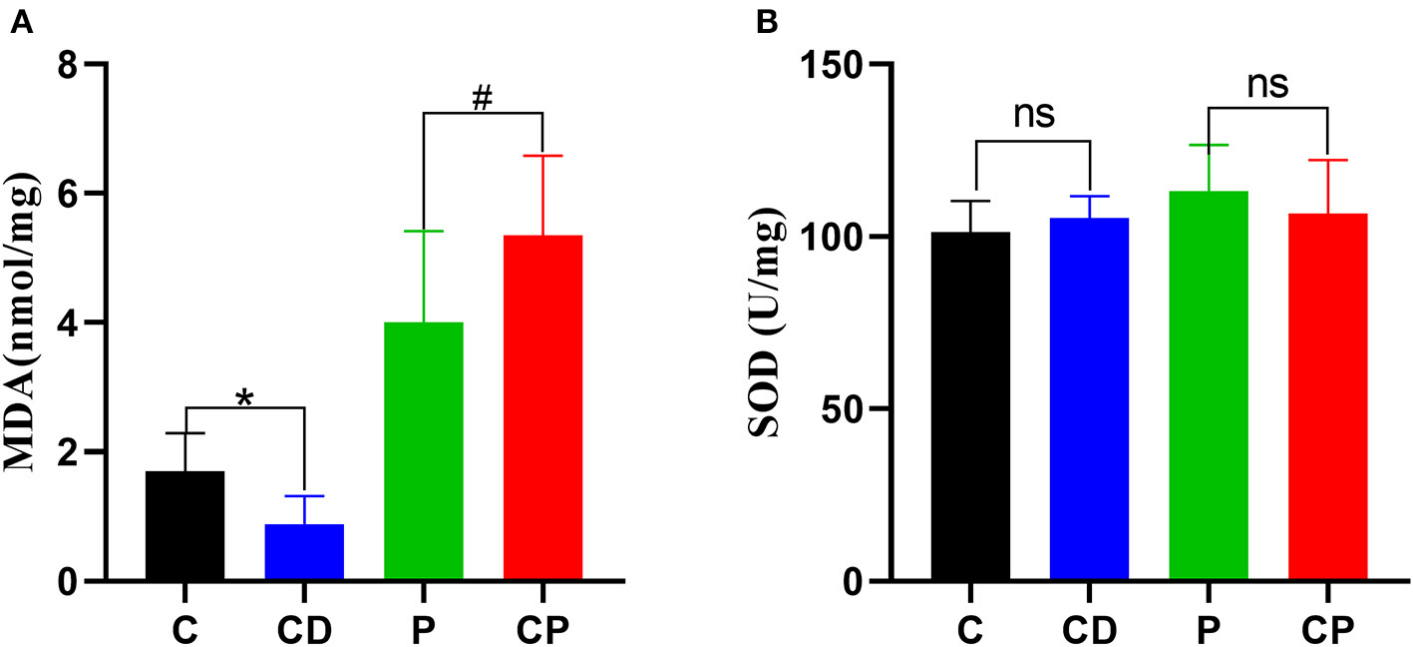
The content of hepatic malonaldehyde (MDA) **(A)** and superoxide dismutase (SOD) **(B)** in liver (*n* = 8–10). * *P* < 0.05; #*P* < 0.05; and ns *P* > 0.05.

In order to test the antioxidant capacity of each group, the MDA index and SOD index of the liver were detected. The content of MDA was decreased in the CD group compared to the C group, while the content of MDA in the CP group was 1.35 nmol/mg higher than that of the P group (*P* < 0.05) (Figure 4). The activity of SOD was increased marginally in the CD group compared to the C group (*P* > 0.05).

### *Chenpi* Modulated the Structural Composition of Intestinal Microbiota

Intestinal microbiota were known as a key factor in modulating obesity. Thus, to investigate whether *chenpi* influences the intestinal microbiota of mice, 16S rRNA sequencing was tested. We analyzed the composition and difference of intestinal microflora in different diet groups.

**Figure 5:**
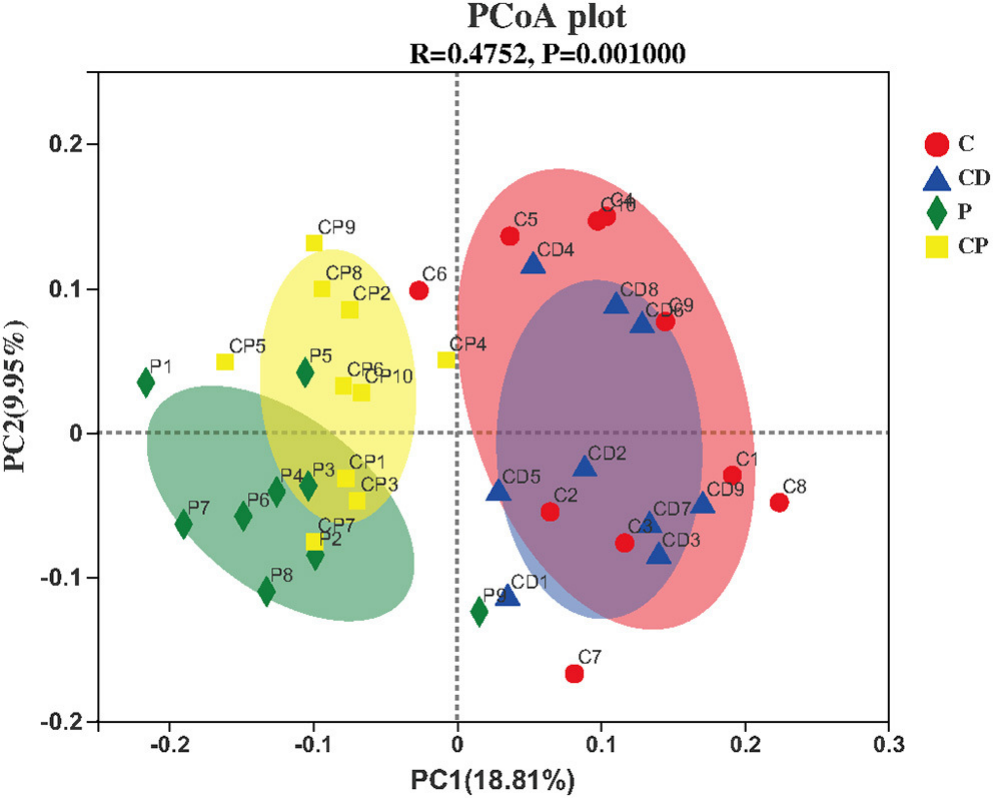
Principal coordinate analysis (PCoA) and plot analysis of different treatment groups (*n* = 10).

**Figure 6:**
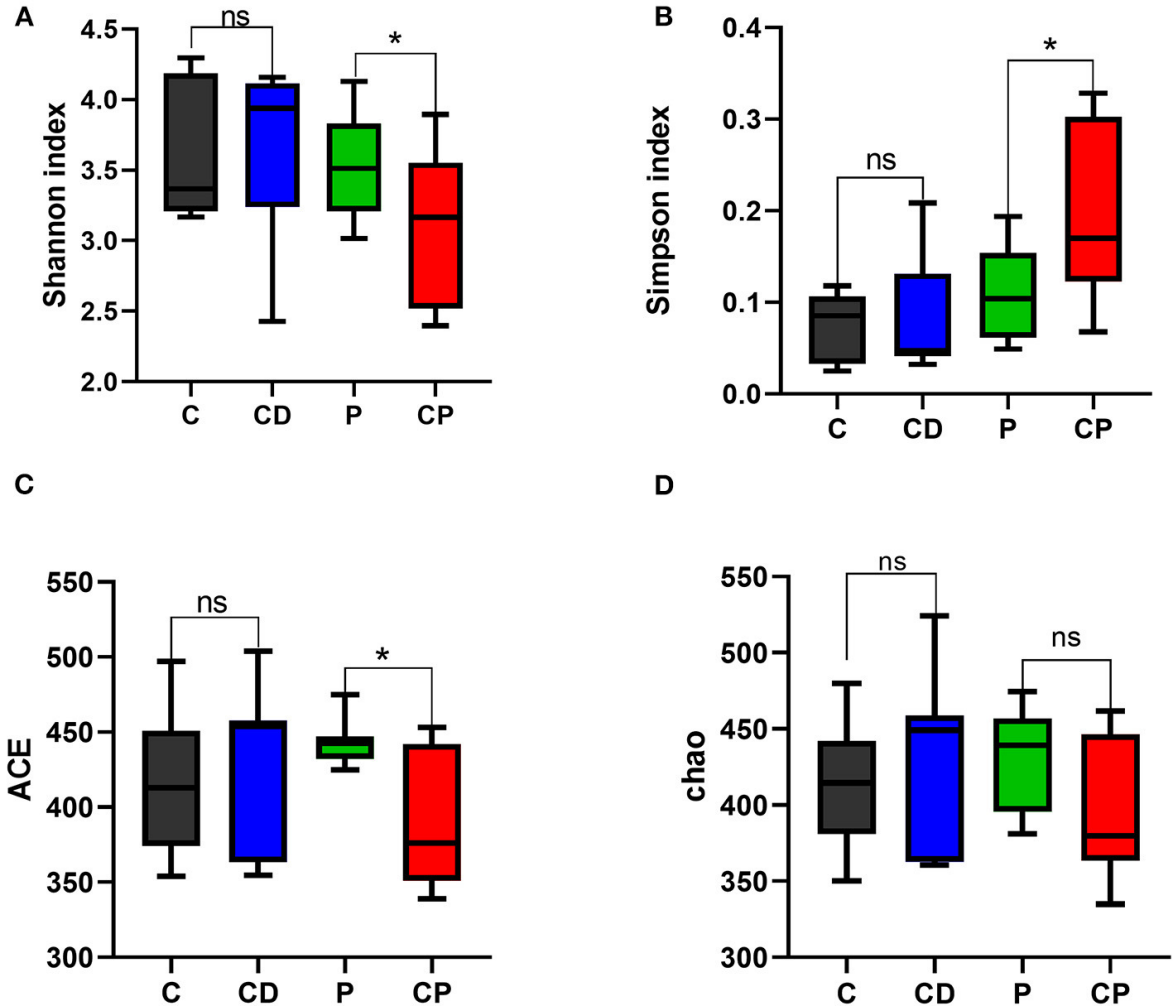
Shannon **(A)**, Simpson's **(B)**, ACE **(C)**, and Chao **(D)** indexes in α-diversity analysis. * *P* < 0.05; ns *P* > 0.05 (*n* = 10).

Microbial diversity and richness were evaluated by α-diversity and β-diversity. PCoA plot was applied to evaluate overall differences in β-diversity in unweighted UniFrac distance for the sample set (34, 35). As shown in Figure 5, different diets have strong effects on the gut microbial composition revealed by a clear separation among four groups. Shannon and Simpson's indexes evaluated the diversity of the microbiota. ACE and Chao indexes described the richness of the microbiota (36). As shown in Figure 6, the CD group exhibited a higher richness of microbiota evidenced by the increased ACE and Chao indexes compared to C (*P* > 0.05) (Figures 6C,D) but with no significant difference. Simpson's index in the CP group significantly increased, but other indexes reduced.

**Figure 7:**
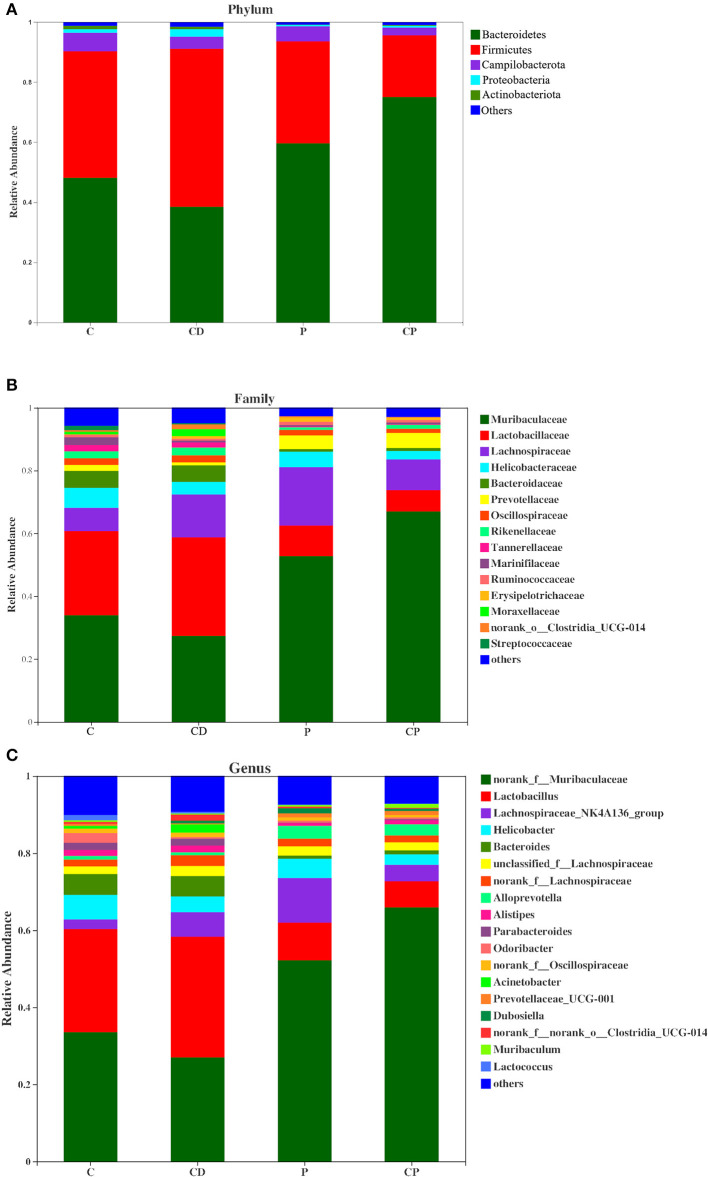
Compositions of microbiota at the phylum **(A)**, family **(B)**, and genus levels **(C)** (*n* = 10).

**Figure 8:**
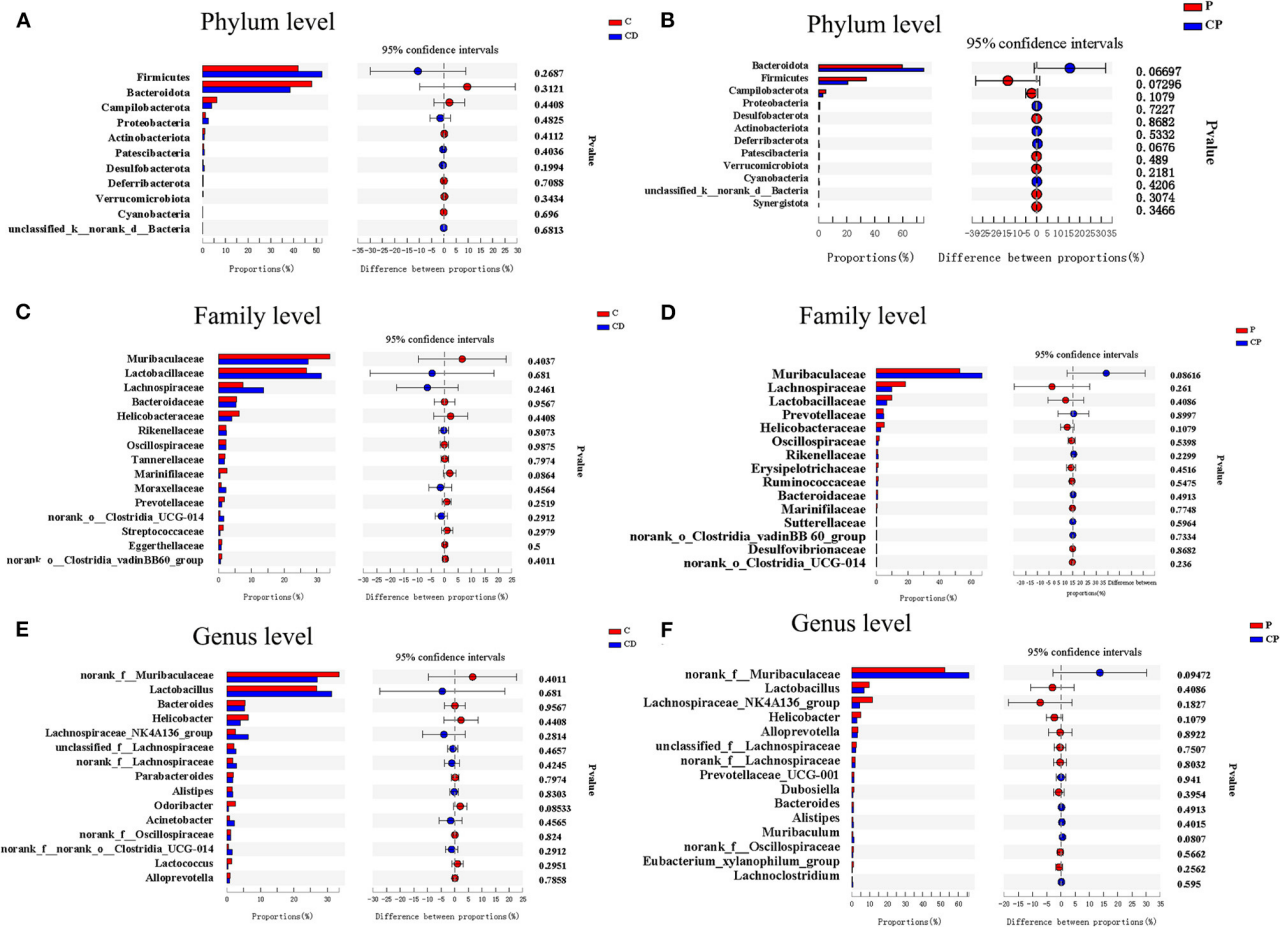
Bar plots of Welch's *t*-test at the phylum, family, and genus levels (*n* = 9–10). **(A)** C group and CD group at the phylum level. **(B)** P group and CP group at the phylum level. **(C)** C group and CD group at the family level. **(D)** P group and CP group at the family level. **(E)** C group and CD group at the genus level. **(F)** P group and CP group at the genus level. *P < 0.05.

As shown in Figure 7, there were differences in microbial composition among the four groups at phylum, family, and genus levels. *Firmicutes* and *Bacteroidetes* are the two majorities at the phylum level. CD group had a 51% higher ratio of *Firmicutes* to *Bacteroidetes* compared with the C group (*P* > 0.05) (Figures 7A, 8A). However, the CP group had a lower abundance of *Firmicutes* (*P* = 0.07) and a higher abundance of *Bacteroidetes* (*P* = 0.06) compared with the P group (Figures 7A, 8B). The difference is not significant. The dominant genera are *Muribaculaceae, Lactobacillaceae*, and *Lachnobacterium* at the family level. The relative abundance of *Lactobacillaceae* in the CD group is higher than in the C group (*p* > 0.05) (Figures 7B, 8C). The relative abundance of *Muribaculaceae* in two powder groups is higher than in two decoction groups (Figure 7B). The relative abundance of *Muribaculaceae* increased in the CP group compared with the P group (*P* = 0.086) (Figures 7B, 8D). Similar alterations were observed for *norank_f_Muribaculaceae, Lactobacillus*, and *Lachnospiraceae _NK4A316_group* at the genus level (Figures 7C, 8E). The relative abundance of *Muribaculaceae* (*p* = 0.09) and *Muribaculum* increased in the CP group compared with the P group (*P* = 0.08) (Figure 8F).

### *Chenpi* Changed the Content of SCFAs in Feces

**Figure 9:**
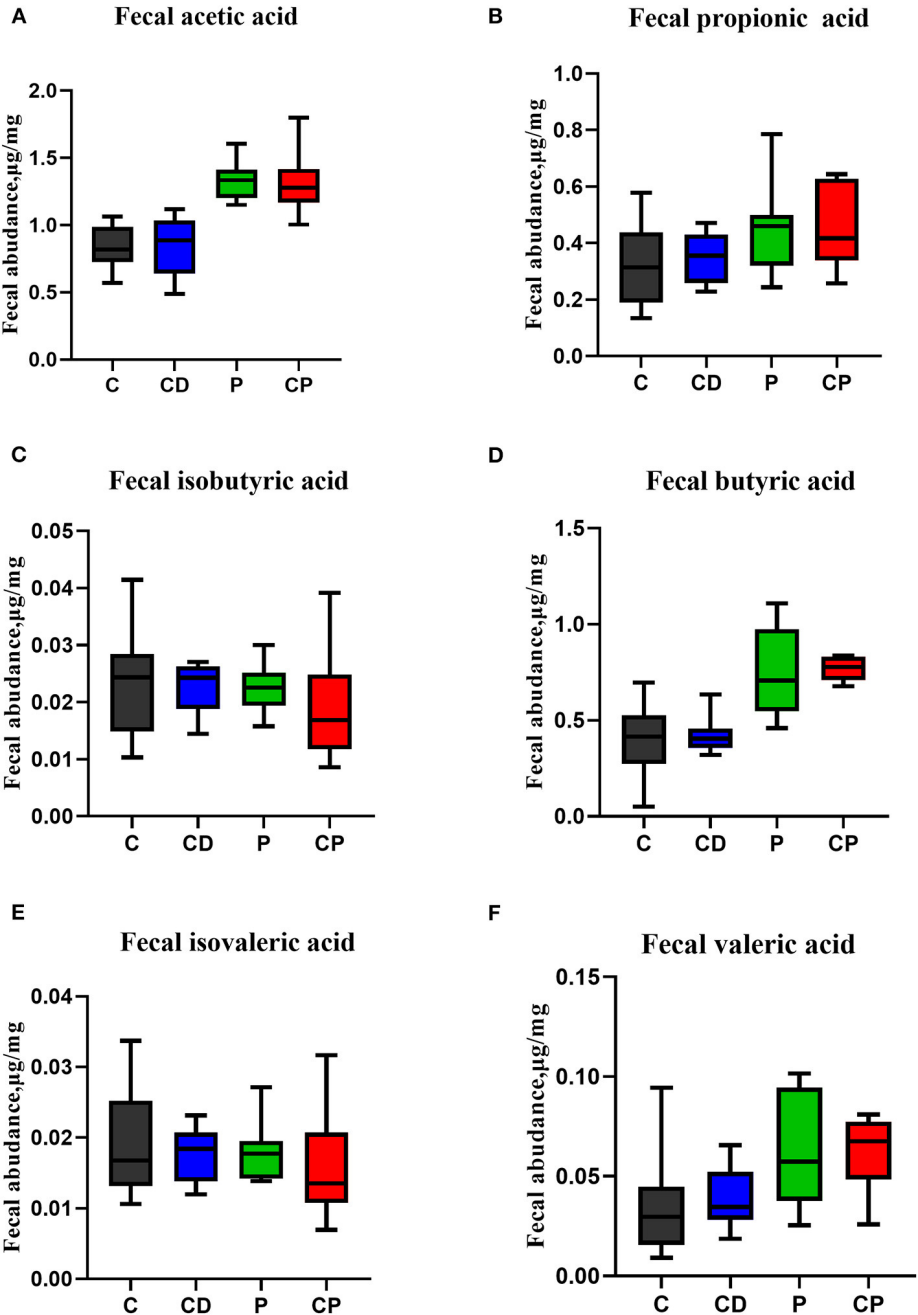
The effect of chenpi on short-chain fatty acids (SCFAs) in the feces (*n* = 9–10). **(A)** Acetic acid, **(B)** propionic acid, **(C)** isobutyric acid, **(D)** butyric acid, **(E)** isovaleric acid, and **(F)** valeric acid.

The content of SCFAs of feces is closely related to intestinal health. Here, the contents of acetic, propionic, butyric, valeric, isobutyric, and isovaleric acids were tested by GC/MSD. On the whole, the content of SCFAs in the two powder groups was higher than that in the decoction groups. There was no difference in the concentration of any SCFAs in feces in the CD group when compared with the control group. The group that consumed *chenpi* powder had higher concentrations of SCFAs than the P group, especially acetic, valeric, and butyric acids, but the difference was not significant (Figure 9).

## Discussion

We present the results of a study investigating the effects of different supplementation treatments with *chenpi* on various health parameters, microbial composition, and content of SCFAs. In healthy mice, *chenpi* supplement changed the accumulation of fat. In particular, *chenpi* powder can effectively reduce the weight of abdominal adipose tissues, subcutaneous adipose tissue, and the serum level of TG. Other studies also have shown that *chenpi* can reduce the gain of body weight, organ weight, and accumulation of lipid (37). Obesity is closely related to hyperlipidemia, and reducing the content of serum triglyceride can effectively alleviate hyperlipidemia (38). There was no significant change in body weight and liver weight in our study, perhaps because the feeding time was not long enough. The effect of *chenpi* on the antioxidant activity of the liver was analyzed. MDA is the most frequently measured biomarkers of lipid peroxidation and oxidative stress that is considered hazardous to health (39). Oxidative damage can lead to a decrease in the content of SOD (40, 41). Here, the decoction of *chenpi* shows stronger antioxidant activity, which might be explained as more antioxidants are released from *chenpi* after decoction treatment using a high temperature (42).

*Chenpi* and its main active substances can affect the composition and richness of intestinal microorganisms. Hesperidin can increase the proportion of *Lactobacillus* in healthy mice. Citrus polymethoxyflavones can greatly enrich the bacterium *Bacteroides* in high-fat diet (HFD) mice (43–45). The abundance of *Proteobacteria* and the ratio of *Firmicutes* to *Bacteroidetes* were decreased by the *chenpi* extract in HFD mice. Although the addition of *chenpi* supplement did not significantly increase the abundance and diversity of intestinal microbiota in our study, it shows that *chenpi* powder has a better tendency to increase the ratio of *Bacteroidetes* to *Firmicutes*. This may be because the decoction does not extract the active ingredients of *chenpi* very well and contains fewer ingredients than *chenpi* powder. Although active compounds such as hesperidin, naringenin, and nobiletin can be detected in the water decoction of *chenpi*, some components cannot be fully and effectively extracted because of their poor water solubility (46). A study showed that the water solubility of 5-demethylnobiletin and hesperidin in *chenpi* was low (47, 48). ACE and Chao indexes reduced in the CP group. This may be related to the reduction in harmful bacteria. Studies show that the abundance of *Bacteroidetes* was reduced by 50%, but *Firmicutes* was increased about 18% (49, 50), the abundance ratio of *Bacteroidetes* to *Firmicutes* will decrease in fat mice compared to lean mice (51, 52), and our results are consistent with them. In the control group, *Muribaculaceae, Lactobacillaceae*, and *Lachnospiraceae* are the dominant strain at the family level. *Chenpi* powder increased the abundance of *Muribaculaceae* significantly at family and genus levels. A high abundance of *Muribaculaceae* is associated with longevity in mice (53). *Chenpi* decoction can increase the abundance of *Lactobacillaceae* that are intestinal beneficial bacteria (54). It has correlation coefficients between bacterial abundances and serum lipid oxidative. The correlations between the abundance of *Lactobacillaceae*, serum TG, and MDA levels were negative (55). *Chenpi* increased intestinal beneficial bacteria and reduced microbial abundance associated with obesity. *Chenpi* powder is more outstanding in the regulation of intestinal microbiota.

The content of SCFAs is closely related to the diet structure. *Chenpi* contains not only many active ingredients but also a lot of dietary fiber. A fiber-rich diet can increase the content of SCFAs in mice. Dietary fiber can be fermented by colonic microbiota to produce SCFAs. Many studies have shown that a diet rich in dietary fiber can change the content of SCFAs. Passion fruit peel can increase the concentrations of butyrate and acetate in cecal content (56). Salami with citrus fiber increased the production of acetate, propionate, and butyrate (57). Dietary fibers from papayas promoted the production of SCFAs (58). Intestinal microorganisms are closely related to SCFAs. *Lachnospiraceae* plays an important role in the production of butyrate (59, 60). *Muribaculaceae* are helpful to the production of propionate (61). *Escherichia coli* could produce acetic acid (62). No significant changes in SCFAs were observed in our study, perhaps due to our shorter feeding cycle.

In conclusion, daily consumption of *chenpi* has a certain effect on reducing weight and lipid. Compared with *chenpi* decoction, *chenpi* powder has a better effect in preventing obesity. *Chenpi* powder may be developed as supplementary functional food to prevent obesity in the future. In this study, we focused on the effect of different treatment methods of *chenpi* on healthy mice to predict the preventive effect on obesity. In the future, a high-fat model would be established to observe this effect in depth. It is our next direction to research study to develop a variety of popular *chenpi* functional foods.

## Data Availability Statement

The datasets presented in this study can be found in online repositories. The names of the repository/repositories and accession number(s) can be found below: PRJNA729616.

## Ethics Statement

The animal study was reviewed and approved by Animal Care and Use Committee of Hunan Agriculture University.

## Author Contributions

JG: Conceptualization. YQ: methodology. YQ and JM: software. YQ, YS, and JG: writing-review and editing. YQ, CW, and ZG: visualization. GL, FF, and YS: supervision. YS: project administration and funding acquisition. All authors contributed to the article and approved the submitted version.

## Conflict of Interest

The authors declare that the research was conducted in the absence of any commercial or financial relationships that could be construed as a potential conflict of interest.

## Publisher's Note

All claims expressed in this article are solely those of the authors and do not necessarily represent those of their affiliated organizations, or those of the publisher, the editors and the reviewers. Any product that may be evaluated in this article, or claim that may be made by its manufacturer, is not guaranteed or endorsed by the publisher.

## References

[1] Tranchida PQ Bonaccorsi I Dugo P Mondello L Dugo G. Analysis of citrus essential oils: state of the art and future perspectives. A review Flavour and Fragr J. (2012) 27:98–123. 10.1002/ffj.2089

[2] Huang R Zhang Y Shen S Zhi Z Cheng H Chen S. Antioxidant and pancreatic lipase inhibitory effects of flavonoids from different citrus peel extracts: An in vitro study. Food Chem. (2020) 326:126785. 10.1016/j.foodchem.2020.126785 32438224

[3] Tian CH Xu H Li J Han Z. Characteristics and intestinal immunomodulating activities of water-soluble pectic polysaccharides from *Chenpi* with different storage periods. Sci Food Agri. (2018) 98:3752–7. 10.1002/jsfa.8888 29330852

[4] Huang J Liao J Qi J Jiang W Yang X. Structural and physicochemical properties of pectin-rich dietary fiber prepared from citrus peel. Food Hydrocoll. (2020) 110:106140. 10.1016/j.foodhyd.2020.106140

[5] Feng K Zhu X Liu G Kan Q Chen T Chen Y. Dietary citrus peel essential oil ameliorates hypercholesterolemia and hepatic steatosis by modulating lipid and cholesterol homeostasis. Food Funct. (2020) 11:7217–30. 10.1039/D0FO00810A 32760938

[6] Raspo AM Vignola BM Andreatta AE Juliani HR. Antioxidant and antimicrobial activity of citrus essential oils from Argentina and the United States. Food Biosci. (2020) 36:100651. 10.1016/j.fbio.2020.100651

[7] Gao Z Wang ZY Guo Y Chu C Zheng GD Liu EH. Enrichment of polymethoxyflavones from *Citrus reticulata* ‘Chachi’ peel and their hypoglycaemic effect. J Chromatogr B. (2019) 1124:226–32. 10.1016/j.jchromb.2019.06.010 31233943

[8] Guo J. TaoH, Cao Y, Ho CT, Jin S. Huang Q prevention of obesity and type 2 diabetes with aged citrus peel (Chenpi) extract. J Agric Food Chem. (2016) 64:2053–61. 10.1021/acs.jafc.5b06157 26912037

[9] Xu JJ Wu X Li MM Li GQ Yang YT Luo HJ. Antiviral activity of polymethoxylated flavones from “guang*Chenpi*”, the edible and medicinal pericarps of citrus reticulata 'chachi'. J. Agric. Food Chem. (2014) 62:2182–2189. 10.1021/jf404310y 24377463

[10] Ling Y Shi Z Y X Cai Z Wang L Wu X. Hypolipidemic effect of pure total flavonoids from peel of Citrus (PTFC) on hamsters of hyperlipidemia and its potential mechanism. Exp Gerontol. (2020) 130:110786. 10.1016/j.exger.2019.110786 31760082

[11] Fu M Zou B An K Yu Y Tang D Wu J. Anti-asthmatic activity of alkaloid compounds from *Pericarpium Citri Reticulatae* (*Citrus reticulata* ‘Chachi’). Food Funct. (2019) 10:903–11. 10.1039/C8FO01753K 30694283

[12] Gunaranjan P Christine AB Halina S Thanuja DH John AM. Short-term feeding of fermentable dietary fibres influences the gut microbiota composition and metabolic activity in rats. Int J Food Sci Tech. (2017) 52:2572–81. 10.1111/ijfs.13543

[13] Macfarlane GT Macfarlane S. Fermentation in the human large intestine: its physiologic consequences and the potential contribution of prebiotics. J Clin Gastroenterol. (2011) 45:S120–7. 10.1097/MCG.0b013e31822fecfe 21992950

[14] Leigh SJ Morris MJ. Diet, inflammation and the gut microbiome: mechanisms for obesity-associated cognitive impairment. Biochim Biophys Acta Mol Basis Dis. (2020) 1866:15767. 10.1016/j.bbadis.2020.165767 32171891

[15] Goyal D Ali SA Singh RK. Emerging role of gut microbiota in modulation of neuroinflammation and neurodegeneration with emphasis on Alzheimer's disease. Prog Neuropsychopharmacol Biol Psychiatry. (2020) 106:110112. 10.1016/j.pnpbp.2020.110112 32949638

[16] Ragonnaud E Biragyn A. Gut microbiota as the key controllers of “healthy” aging of elderly people. Immun Ageing. (2021) 18:2. 10.1186/s12979-020-00213-w 33397404 PMC7784378

[17] Xia Y Tan D Akbary R Kong J Seviour R Kong Y. Aqueous raw and ripe Pu-erh tea extracts alleviate obesity and alter cecal microbiota composition and function in diet-induced obese rats. Appl Microbiol Biotechnol. (2019) 103:1823–35. 10.1007/s00253-018-09581-2 30610284

[18] Lee ES Song EJ Nam YD Nam TG Kim HJ Lee BH. Effects of enzymatically modified chestnut starch on the gut microbiome, microbial metabolome, and transcriptome of diet induced obese mice. Int J Bio Macromolecules. (2019) 145:245–243. 10.1016/j.ijbiomac.2019.12.169 31870873

[19] Liu M Li X Zhou S Wang TTY Zhou S Yang K. Dietary fiber isolated from sweet potato residues promote healthy gut microbiome profile. Food Funct. (2020) 11:689–99. 10.1039/C9FO01009B 31909777

[20] Zhang M Zhu J Zhang X Zhao D Ma Y Li D. Aged citrus peel (*Chenpi*) extract causes dynamic alteration of colonic microbiota in high-fat diet induced obese mice. Food Funct. (2020) 11:2667–78. 10.1039/C9FO02907A 32159537

[21] Tung YC Chang WT Li S Wu JC Bademeav V Ho CT. Citrus peel extracts attenuated obesity and modulated gut microbiota in a high-fat diet-induced obesity mice. Food Funct. (2018) 9:3363–73. 10.1039/C7FO02066J 29855643

[22] Yu M Li Z Chen W Wang G Cui Y Ma X. Dietary supplementation with citrus extract altered the intestinal microbiota and microbial metabolite profiles and enhanced the mucosal immune homeostasis in yellow-feathered broilers. Front in microbiol. (2019) 10:2662. 10.3389/fmicb.2019.02662 31849855 PMC6887900

[23] Paavonsalo S Hariharan S Lackman MH Karaman S. Capillary rarefaction in obesity and metabolic diseases-organ-specificity and possible mechanisms. Cells. (2020) 9:2683. 10.3390/cells9122683 33327460 PMC7764934

[24] Gesta S H Y Tseng Kahn CR. Developmental origin of fat: tracking obesity to its source. Cell. (2008) 135:366. 10.1016/j.cell.2008.09.048 17956727

[25] Shtriker MG Hahn M Taieb E Nyska A Moallem U Tirosh O. Fenugreek galactomannan and citrus pectin improve several parameters associated with glucose metabolism and modulate gut microbiota in mice. Nutrition. (2018) 134–42. 10.1016/j.nut.2017.07.012 28993009

[26] Liu X Martin DA Valdez JC Sudakaran S Rey F Bolling BW. Aronia berry polyphenols have matrix-dependent effects on the gut microbiota. Food Chem. (2021) 359:129831. 10.1016/j.foodchem.2021.129831 33957324

[27] Hu Y Hou Z Yi R Wang Z Sun P Li G. Tartary buckwheat flavonoids ameliorate high fructose-induced insulin resistance and oxidative stress associated with the insulin signaling and Nrf2/HO-1 pathways in mice. Food Funct. (2017) 8:2803–16. 10.1039/C7FO00359E 28714504

[28] Yin J Li Y Han H Chen S Gao J Liu G. Melatonin reprogramming of gut microbiota improves lipid dysmetabolism in high-fat diet-fed mice. J Pineal Res. (2018) 65:e12524. 10.1111/jpi.12524 30230594

[29] Chen LC Fan ZY Wang HY Wen DC Zhang SY. Effect of polysaccharides from adlay seed on anti-diabetic and gut microbiota. Food Funct. (2019) 10:4372–80. 10.1039/C9FO00406H 31276140

[30] Fan L Qi Y Qu S Chen X Li A Hendi M. B. adolescentis ameliorates chronic colitis by regulating Treg/Th2 response and gut microbiota remodeling. Gut Microbes. (2021) 13:1–17. 10.1080/19490976.2020.1826746 33557671 PMC7889144

[31] Ye M Sun J Chen Y Ren Q Li Z Zhao Y. Oatmeal induced gut microbiota alteration and its relationship with improved lipid profiles: a secondary analysis of a randomized clinical trial. Nutr Metab (Lond). (2020) 17:85. 10.1186/s12986-020-00505-4 33042205 PMC7542720

[32] Xiao S Liu C Chen M Zou J Zhang Z Cui X. Scutellariae radix and coptidis rhizoma ameliorate glycolipid metabolism of type 2 diabetic rats by modulating gut microbiota and its metabolites. Appl Microbiol Biotechnol. (2020) 104:303–17. 10.1007/s00253-019-10174-w 31758238

[33] Tamura K Sasaki H Shiga K Miyakawa H Shibata S. The timing effects of soy protein intake on mice gut microbiota. Nutrients. (2019) 12:87. 10.3390/nu12010087 31892229 PMC7019473

[34] Yin J Han H Li Y Liu Z Zeng X Li T. Lysine restriction affects feed intake and amino acid metabolism via gut microbiome in piglets. Cell Physiol Biochem. (2017) 44:1749–61. 10.1159/000485782 29216634

[35] Duan Y Zhong Y Xiao H Zheng C Song B Wang W. Gut microbiota mediates the protective effects of dietary beta-hydroxy-beta-methylbutyrate (HMB) against obesity induced by high-fat diets. FASEB J. (2019) 33:10019–33. 10.1096/fj.201900665RR 31167080

[36] Van den Brule S Rappe M Ambroise J Bouzin C Dessy C Paquot A. Diesel exhaust particles alter the profile and function of the gut microbiota upon subchronic oral administration in mice. Part Fibre Toxicol. (2021) 18:7. 10.1186/s12989-021-00400-7 33563307 PMC7871568

[37] Guo J Cao Y Ho C-T Jin S Huang Q. Aged citrus peel (chenpi) extract reduces lipogenesis in differentiating 3T3-L1 adipocytes. J Funct Foods. (2017) 34:297–303. 10.1016/j.jff.2017.04.042

[38] Chen K Ma Z Yan X Liu J Xu W Li Y. Investigation of the lipid-lowering mechanisms and active ingredients of danhe granule on hyperlipidemia based on systems pharmacology. Front Pharmacol. (2020) 11:528. 10.3389/fphar.2020.00528 32435189 PMC7218108

[39] Tsikas D. Assessment of lipid peroxidation by measuring malondialdehyde (MDA) and relatives in biological samples: Analytical and biological challenges. Anal Biochem. (2017) 524:13–30. 10.1016/j.ab.2016.10.021 27789233

[40] Zhang C Zhao J Famous E Pan S Peng X. Tian J. Antioxidant, hepatoprotective and antifungal activities of black pepper (Piper nigrum L) essential oil. Food Chem. (2021) 346:128845. 10.1016/j.foodchem.2020.128845 33387832

[41] Wu Q Li W Zhao J Sun W Yang Q Chen C. Apigenin ameliorates doxorubicin-induced renal injury via inhibition of oxidative stress and inflammation. Biomed Pharmacother. (2021) 137:111308. 10.1016/j.biopha.2021.111308 33556877

[42] Choi MY Chai C Park JH Lim J Lee J Kwon SW. Effects of storage period and heat treatment on phenolic compound composition in dried Citrus peels (Chenpi) and discrimination of Chenpi with different storage periods through targeted metabolomic study using HPLC-DAD analysis. J Pharm Biomed Anal. (2011) 54:638–45. 10.1016/j.jpba.2010.09.036 21145683

[43] Stevens Y Rymenant EV Grootaert C Camp JV Possemiers S Masclee A. The intestinal fate of citrus flavanones and their effects on gastrointestinal health. Nutrients. (2019) 11:1464. 10.3390/nu11071464 31252646 PMC6683056

[44] Estruel-Amades S Massot-Cladera M Perez-Cano FJ Franch A Castell M Camps-Bossacoma M. Hesperidin effects on gut microbiota and gut-associated lymphoid tissue in healthy rats. Nutrients. (2019) 11:324. 10.3390/nu11020324 30717392 PMC6412496

[45] Zeng SL Li SZ Xiao PT Cia YY Chu C Chen BZ. Citrus polymethoxyflavones attenuate metabolic syndrome by regulating gut microbiome and amino acid metabolism. Sci Adv. (2020) 6:eaax6208. 10.1126/sciadv.aax6208 31922003 PMC6941918

[46] Cao R Zhao Y Zhou Z Zhao X. Enhancement of the water solubility and antioxidant activity of hesperidin by chitooligosaccharide. J Sci Food Agric. (2018) 98:2422–7. 10.1002/jsfa.8734 29023808

[47] Ning F Wang X Zheng H Zhang K Bai C Peng H. Improving the bioaccessibility and in vitro absorption of 5-demethylnobiletin from chenpi by se-enriched peanut protein nanoparticles-stabilized pickering emulsion. J Funct Foods. (2019) 55:76–85. 10.1016/j.jff.2019.02.019

[48] Zhang Y Yu Y Li H Huang W Wang P. Effects of Citri Reticulatae Pericarpium and grapefruit juice on the pharmacokinetics of omeprazole in rats. J Food Bio. (2021) 00:e13804. 10.1111/jfbc.13804 34080214

[49] Louis P Flint HJ. Formation of propionate and butyrate by the human colonic microbiota. Environ Microbiol. (2017) 19:29–41. 10.1111/1462-2920.13589 27928878

[50] Ley RE Backhed F Turnbaugh P Lozupone CA Knight RD Gordon JI. Obesity alters gut microbial ecology. Proc Natl Acad Sci USA. (2005) 102:11070–11075. 10.1073/pnas.0504978102 16033867 PMC1176910

[51] Long JF Yang JP Henning SM Woob SL Hsu M. ChanB. Xylooligosaccharide supplementation decreases visceral fat accumulation and modulates cecum microbiome in mice. J Funct Foods. (2019) 52:138–46. 10.1016/j.jff.2018.10.035

[52] Evans CC LePard KJ Kwak JW Stancukas MC Laskowski S Dougherty J. Exercise prevents weight gain and alters the gut microbiota in a mouse model of high fat diet-induced obesity. PLoS ONE. (2014) 9:e92193. 10.1371/journal.pone.0092193 24670791 PMC3966766

[53] Sibai M Altuntas E Yildirim B Ozturk G Yildirim S Demircan T. Microbiome and longevity: high abundance of longevity-linked muribaculaceae in the gut of the long-living rodent spalax leucodon. Omics: a Journal of Integrative Biology. (2020) 24:592–601. 10.1089/omi.2020.0116 32907488

[54] Liu H Zhu H Xia H Yang X Yang L Wang S. Different effects of high-fat diets rich in different oils on lipids metabolism, oxidative stress and gut microbiota. Food Res Int. (2020) 141:110078. 10.1016/j.foodres.2020.110078 33641963

[55] Ojo BA O'Hara C Wu L El-Rassi D Ritchey JW Chowanadisai W. Wheat germ supplementation increases lactobacillaceae and promotes an anti-inflammatory gut milieu in C57BL/6 mice fed a high-fat, high-sucrose diet. J Nutr. (2019) 149:1107–15. 10.1093/jn/nxz061 31162575

[56] Da Silva JK Cazarin CBB Bogusz Junior S Augusto F Maróstica Junior MR. Passion fruit (Passiflora edulis) peel increases colonic production of short-chain fatty acids in Wistar rats. LWT - Food Sci Tech. (2014) 59:1252–7. 10.1016/j.lwt.2014.05.030

[57] Pérez-Burillo S Mehta T Pastoriza S Kramer DL Paliy O Rufian-Henares JA. Potential probiotic salami with dietary fiber modulates antioxidant capacity, short chain fatty acid production and gut microbiota community structure. Lebensm Wiss Technol. (2019) 105:355–62. 10.1016/j.lwt.2019.02.006

[58] Do Prado SBR Minguzzi BT Hoffmann C Fabi JP. Modulation of human gut microbiota by dietary fibers from unripe and ripe papayas: Distinct polysaccharide degradation using a colonic in vitro fermentation model. Food Chem. (2021) 348:129071. 10.1016/j.foodchem.2021.129071 33493843

[59] Rinninella E Raoul P Cintoni M Franceschi F Miggiano GAD Gasbarrini A. What is the healthy gut microbiota composition? a changing ecosystem across age, environment, diet, and diseases. Microorganisms. (2019) 7:14. 10.3390/microorganisms7010014 30634578 PMC6351938

[60] Berger K Burleigh S Lindahl M Bhattacharya A Patil P Stalbrand H. Xylooligosaccharides increase bifidobacteria and lachnospiraceae in mice on a high-fat diet, with a concomitant increase in short-chain fatty acids, especially butyric acid. J Agric Food Chem. (2021) 69:3617–25. 10.1021/acs.jafc.0c06279 33724030 PMC8041301

[61] Smith BJ Miller RA Ericsson AC Harrison DC Strong R Schmidt TM. Changes in the gut microbiome and fermentation products concurrent with enhanced longevity in acarbose-treated mice. BMC Microbiol. (2019) 19:130. 10.1186/s12866-019-1494-7 31195972 PMC6567620

[62] Nakkarach A Foo HL Song AA Mutalib NEA Nitisinprasert S Withayagiat U. Anti-cancer and anti-inflammatory effects elicited by short chain fatty acids produced by Escherichia coli isolated from healthy human gut microbiota. Microb Cell Fact. (2021) 20:36. 10.1186/s12934-020-01477-z 33546705 PMC7863513

